# Molecular epidemiology of carbapenem resistant *Enterobacteriaceae* in Valle d’Aosta region, Italy, shows the emergence of KPC-2 producing *Klebsiella pneumoniae* clonal complex 101 (ST101 and ST1789)

**DOI:** 10.1186/s12866-015-0597-z

**Published:** 2015-11-09

**Authors:** Mariateresa Del Franco, Laura Paone, Roberto Novati, Claudio G. Giacomazzi, Maria Bagattini, Chiara Galotto, Pier Giorgio Montanera, Maria Triassi, Raffaele Zarrilli

**Affiliations:** Department of Public Health, University of Naples ‘Federico II’, Naples, Italy; Medical Direction, Aosta Regional Hospital, Aosta, Italy; Microbiology, Aosta Regional Hospital, Aosta, Italy; CEINGE Biotecnologie Avanzate, Naples, Italy

**Keywords:** Epidemiological typing, Carbapenem resistant *Enterobacteriaceae*, Carbapenemase, Horizontal gene transfer

## Abstract

**Background:**

The spread of carbapenem resistant *Enterobacteriaceae* (CRE) is an emerging clinical problem, of great relevance in Europe and worldwide. The aim of this study was the molecular epidemiology of CRE isolates in Valle d’Aosta region, Italy, and the mechanism of carbapenem resistance.

**Results:**

Sixty consecutive CRE samples were isolated from 52 hospital inpatients and/or outpatients from November 2013 to August 2014. Genotyping of microbial isolates was done by pulsed-field gel electrophoresis (PFGE) and multi-locus sequence typing (MLST), carbapenemases were identified by PCR and sequencing. Carbapenem resistance gene transfer was performed by filter mating, plasmids from parental and transconjugant strains were assigned to incompatibility groups by PCR-based replicon typing. Molecular characterization of CRE isolates assigned 25 *Klebsiella pneumoniae* isolates to PFGE types A1-A5 and sequencing type (ST) 101, 17 *K. pneumoniae* isolates to PFGE type A and ST1789 (a single locus variant of ST101), 7 *K. pneumoniae* isolates to PFGE types B or C and ST512, 2 *K. pneumoniae* isolates to PFGE type D and ST405, and 5 *Escherichia coli* isolates to PFGE type a and ST131. All *K. pneumoniae* ST101 and ST1789 isolates were extended-spectrum beta-lactamase (ESBL) producers and carried *bla*_CTX-M-1 group_ gene; 4 *K. pneumoniae* ST101 isolates were resistant to colistin. Molecular analysis of beta-lactamase genes identified *bla*_KPC-2_ and *bla*_CTX-M-group 1_ into conjugative plasmid/s assigned to IncFII incompatibility group in ST101 and ST1789 *K. pneumoniae* isolates, *bla*_KPC-3_ into conjugative plasmid/s assigned to IncF incompatibility group in ST512 and ST405 *K. pneumoniae* isolates, *bla*_VIM-1_ into conjugative plasmid/s assigned to IncN incompatibility group in ST131 *E. coli* isolates.

**Conclusions:**

The spread of CRE in Valle d’Aosta region was caused by the selection of KPC-2 producing *K. pneumoniae* ST101 and ST1789 epidemic clones belonging to clonal complex 101, KPC-3 producing *K. pneumoniae* epidemic clones assigned to ST512 and ST405, and VIM-1 producing *E.coli* ST131 epidemic clone. Carbapenem resistance, along with *bla*_KPC-2_, *bla*_KPC-3_ and *bla*_VIM-1_ carbapenemase genes, was transferred by conjugative plasmids assigned to IncFII, IncF, and IncN incompatibility groups, respectively, in filter mating experiments. The emergence of colistin resistance was observed in KPC-2 producing *K. pneumoniae* ST101 isolates.

**Electronic supplementary material:**

The online version of this article (doi:10.1186/s12866-015-0597-z) contains supplementary material, which is available to authorized users.

## Background

The spread of resistance to carbapenems among *Enterobacteriaceae* has become a major public health problem. In fact, epidemics or endemic conditions of patients infected or colonized by *Enterobacteriaceae* non susceptible to carbapenems has been reported in the hospital and in the community setting [1–3]. Carbapenem resistant *Enterobacteriaceae* (CRE) are resistant to most β-lactams, including the carbapenems, and frequently carry additional antimicrobial resistance genes to other non-β-lactam antibiotics, which render them resistant to most antibiotics [1–3].

Two main mechanisms are responsible for carbapenem resistance in *Enterobacteriaceae*: reduced outer membrane permeability by porin loss in combination with the production of an extended spectrum beta-lactamase or of AmpC-type beta-lactamase; and the production of beta-lactamases capable of hydrolysing carbapenems (carbapenemases) [1–3]. Because acquired carbapenemases are encoded by transferable genes located in mobile elements such as plasmids and transposons, which may disseminate among different strains and species, carbapenemase-mediated resistance represents the major treat in CRE [1–6]. The occurrence of either class B metallo-beta-lactamases (IMP, VIM, NDM) or classes A (KPC) and D (OXA-48) serine carbapenemases have been reported in Europe and world-wide [1–6]. In Italy, *Enterobacteriaceae* producing VIM-type enzymes and more recently KPC-type enzymes have been described [7–10]. The increase of carbapenem resistance in *Enterobacteriaceae* has been sustained also by the selection of multidrug-resistant high risk clonal lineages, which successfully propagated in the hospital and the community settings [6, 11]. Also, the coexistence of carbapenem resistance and extended-spectrum beta-lactamase (ESBL) production has been frequently described [2, 12–14]. Moreover, the movement of colonized patients, mainly elderly, between hospital wards and long-term care settings may contribute to the spread of CRE [1–3, 8].

To control the spread of CRE in health care facilities and the transmission of CRE among patients, guidelines have been established from European Centre for Disease Prevention and Control for the screening and identification of CRE isolates among colonized patients [15], which have been adopted in Italy, both at regional and national level [16].

The aim of the current study was to analyze the molecular epidemiology of 60 consecutive CRE in the hospital wards and long-term care facilities of the USL Valle d’Aosta, and the mechanism of carbapenem resistance.

## Methods

### Setting and study period

Valle d’Aosta is the smallest region of Italy (3262 sq Kms), entirely located in the north western Alps region; there is one health agency and only one public Hospital, with 480 beds: there is one microbiology laboratory that processes samples from both Hospital and the territory. Moreover, a network of about 45 long-term care residencies for elders is distributed over the territory, accounting for another 1000 beds. The present study analyzed 60 consecutive isolates of CRE from 52 hospital inpatients and/or outpatients who were admitted to the USL Valle d’Aosta between November 2013 and August 2014.

### Surveillance of CRE *Enterobacteriaceae*

Surveillance of CRE was performed according to European Centre for Disease Prevention and Control [15] and the Ministero della Salute, Italy [16]. Surveillance and infection control measures adopted for CRE positive patients included: rectal swab screening of both hospital inpatients and outpatients; isolation and cohorting of CRE positive hospital inpatients; recall and screening of CRE positive outpatients.

### Bacterial strains and microbiological methods

All the *Enterobacteriaceae* isolates were identified using the Vitek 2 automatic system and the ID-GNB card for identification of Gram-negative bacilli according to the manufacturer’s instructions (bio-Merieux, Marcy l’Etoile, France). Two *Raoultella spp.*, and one *Enterobacter spp.* were further identified as *R. planticola, R. ornithinolytica* and *E. aerogenes* by amplification and sequencing of the rpoB gene as previously described [17].

### Antimicrobial susceptibility testing

Carbapenem resistance of *Enterobacteriaceae* was screened using the meropenem disk alone as previously described [18]. Susceptibility tests were performed using the Vitek 2 system and the AST-GN card (bioMe’rieux). Breakpoints values were those recommended by the EUCAST 2014 [19]. *Escherichia coli* ATCC 25922 was used as quality control strain. ESBL activity was evaluated using the double-disk synergy test as previously described [20]. Colistin susceptibility was evaluated by broth microdilution in Mueller–Hinton broth II (MHBII) according to Clinical and Laboratory Standards Institute guidelines [21].

### Molecular analysis of antimicrobial resistance genes

All the isolates were subjected to polymerase chain reaction (PCR) to screen for the presence of *bla*_KPC_, *bla*_IMP_, *bla*_VIM_, *bla*_NDM_ and *bla*_OXA-48_, as described previously [22]. *Acinetobacter baumannii* AC 54/97 [23], *A. baumannii* 161/07 [24] and *Klebsiella pneumoniae* D001 [18] were used as positive quality control strains for *bla*_IMP-2_, *bla*_NDM-1_ and *bla*_OXA-48_ carbapenemase genes, respectively. The presence of the correct size PCR product was confirmed by agarose gel electrophoresis. The full-length alleles of *bla*_KPC_ and *bla*_VIM_ were amplified using primers KPC-F2: GTATCGCCGTCTAGTTCTGC and KPC-Rm: CTTGTCATCCTTGTTAGGCG, primers 5’VIM1: ATGTTAAAAGTTATTAGTAGTT and 3’VIM1: CTACTCGGCGACTGAGCGATT, respectively. Direct sequencing of PCR products was performed using the ABI Prism BigDye Terminator v3.1 Ready Reaction cycle sequencing kit and the 3730 DNA analyzer (Applied Biosystems, Foster City, CA). Nucleotide sequences obtained were compared with the deduced amino acid sequence available in GenBank using tblastx [25]. The presence of *bla*_CTX-M_ extended-spectrum beta-lactamase genes was investigated by multiplex PCR as described previously [26]. Escherichia coli SGH 20-ADM was used as positive quality control strain for *bla*_CTX-M_-_15 gene_ [27]. The presence of the *armA* methylase gene was investigated by PCR using specific primers as described previously [28].

### PFGE typing and dendrogram analysis

*Xba*I DNA macrorestriction of *Enterobacteriaceae* isolates was performed as previously reported [29]. PFGE conditions were as follows: pulse times ranged from 5 to 40 s over 24 h at6 · 0 V/cm and at 14C. The PFGE profiles obtained were converted to TIFF files and subjected to cluster analysis using the GelCompare II v. 4.6 software package (AppliedMaths, Sint-Martens-Latem, Belgium). Clustering was based on the unweighted pair-group method with arithmetic averages (UPGMA). The Dice correlation coefficient was used to analyze the similarities of the banding patterns with a tolerance of 1.5 %. The interpretation of chromosomal DNA restriction patterns was based on the criteria of Tenover et al. for closely related isolates [30]. Briefly, strains showing more than three DNA fragment variations and a similarity of <85 % at dendrogram analysis were considered to represent different PFGE types, while one to three-fragment differences and a similarity of >85 % upon dendrogram analysis were considered to represent PFGE pattern subtypes.

### MLST analysis

MLST analysis of *K. pneumoniae* isolates was performed using the Institut Pasteur’s MLST scheme [31]. The allele sequences and sequence types (STs) were identified at http://bigsdb.web.pasteur.fr/klebsiella/klebsiella.html. MLST analysis of *E. coli* isolates was performed as previously described [32, 33], with the following minor modifications for annealing temperatures of the primers, respectively 54 °C for *adk*, *fumC* and *icd* genes, 58 °C for *recA* and *mdh* genes, 60 °C for *purA* gene, and 64 °C for *gyrB* gene. Allele sequence and MLST profile definitions were identified at http://mlst.warwick.ac.uk/mlst/dbs/Ecoli.

### Conjugative transfer of carbapenem resistance and plasmid typing

Conjugation experiments were performed by filter mating experiments as previously described [34] using sodium-azide resistant *E. coli* J53 [35] as recipient strain on BHI agar plates in the presence of 16 mg/l imipenem and 100 mg/l sodium-azide. The frequency of transfer was calculated as the number of transconjugants divided by the number of surviving recipients. Plasmids from parental and transconjugant strains were assigned to incompatibility groups by PCR-based replicon typing (PBRT-kit) (Diatheva s.r.l., Fano, Italy) performed on total DNA using previously described primers and conditions [36, 37].

### Ethical approval

The study was approved by the ethics committee of the Aosta Regional Hospital (protocol number 836/2015). All microbiological samples were taken as part of standard care procedures. No written informed consent was necessary for this type of study.

## Results

### CRE isolates included in the study

**Fig. 1:**
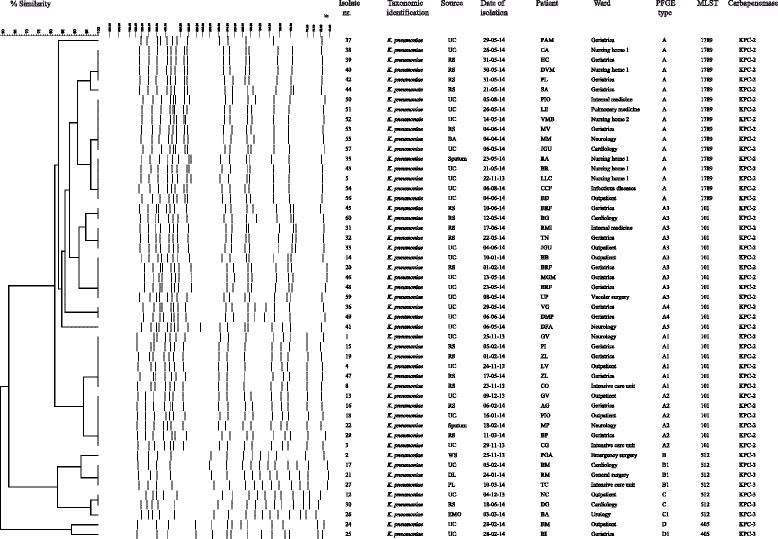
Genotypic analysis of carbapenem resistant *K. pneumoniae* isolates. Dendrogram analysis of digitized *Xba*I PFGE profiles of carbapenem resistant *K. pneumoniae* isolates is shown. Percentage of similarity at dendrogram analysis and sizes in kilobases (kb) of lambda DNA molecular mass markers are indicated above dendrogram and PFGE profiles, respectively. Isolate number, taxonomic identification, source, date of isolation, patient name, ward, PFGE type, MLST sequence type and carbapenemase are shown also. UC, urine culture; RS, rectal swab; WS, wound swab; DL, drainage liquid; EMO, blood culture; PF, peritoneal fluid; BA, bronchial aspirate; PFGE, pulsed-field gel electrophoresis; MLST, multilocus sequence typing; ST, sequence type

**Fig. 2:**
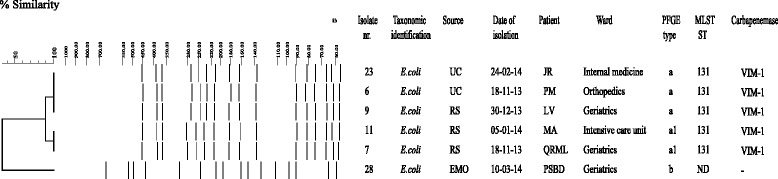
Genotypic analysis of carbapenem resistant *E. coli* isolates. Dendrogram analysis of digitized *Xba*I PFGE profiles of carbapenem resistant *E. coli* isolates is shown. Percentage of similarity at dendrogram analysis and sizes in kilobases (kb) of lambda DNA molecular mass markers are indicated above dendrogram and PFGE profiles, respectively. Isolate number, taxonomic identification, source, date of isolation, patient name, ward, PFGE type, MLST sequence type and carbapenemase are shown also. UC, urine culture; RS, rectal swab; WS, wound swab; DL, drainage liquid; EMO, blood culture; PF, peritoneal fluid; BA, bronchial aspirate; PFGE, pulsed-field gel electrophoresis; MLST, multilocus sequence typing; ST, sequence type

The molecular epidemiology of carbapenem-resistant *Enterobacteriaceae* was studied in the Valle d’Aosta region from November 2013 to August 2014. During this period, 60 consecutive CRE were isolated from 52 different patients, both from clinical samples and from surveillance screening. Of these patients, 6 were outpatients, 4 were admitted to nursing homes, while the remaining 42 were admitted to hospital’s internal wards (in particular, 19 in geriatrics, 4 in intensive care, 4 in neurology, 10 in medical departments and 5 in surgical wards). The isolated materials were 32 urine culture, 20 rectal swabs, 3 respiratory secretions, 2 blood culture, 1 liquid drainage, 1 peritoneal-fluid, and 1 wound swab. Fifty-one isolates were identified as *K. pneumoniae*, 6 were *E. coli*, 1 *R. planticola,* 1 *R. ornithinolytica*, and 1 *E. aerogenes* (Figs. 1 and 2 and Additional file 1: Table S1).

### Antimicrobial susceptibility patterns of *Enterobacteriaceae* isolates

Both *K. pneumoniae* and *E. coli* isolates showed a multidrug-resistant antibiotype. In particular, they were resistant to imipenem, meropenem, ertapenem, beta-lactam/beta-lactamase inhibitor combinations (clavulanic acid/amoxicillin, piperacillin/tazobactam), third and fourth generation cephems, fluoroquinolones (ciprofloxacin), and trimethoprim-sulfamethoxazole; 38 *K. pneumoniae* isolates were resistant but 13 *K. pneumoniae* isolates were susceptible to aminoglycosides (amikacin and gentamicin); 46 *K. pneumoniae* isolates were resistant but 5 *K. pneumoniae* isolates were susceptible to fosfomycin; all *E. coli* isolates were susceptible to aminoglycosides and fosfomycin. Also, 42 *K. pneumoniae* isolates were identified as ESBL producers (Table 1 and Additional file 1: Table S1). Interestingly, all *E. coli* isolates and the majority of *K. pneumoniae* isolates were susceptible to colistin, but 4 *K. pneumoniae* isolates were resistant to colistin, minimal inhibitory concentrations (MICs) 16–125 mg/l (Table 1 and Additional file 1: Table S1). *Raoultella spp* and *E. aerogenes* isolates were resistant to carbapenems, beta-lactam/beta-lactamase inhibitor combinations, third and fourth generation cephems, fluoroquinolones and trimethoprim-sulfamethoxazole but susceptible to aminoglycosides, fosfomycin and colistin (Additional file 1: Table S1).

**Table 1:** Antibiotic susceptibility profiles of carbapenem resistant *K. pneumoniae* and *E. coli*

MIC^a^ (mg/l)						
Antibiotic	*K. pneumoniae* (51 total stains)			*E. coli* (6 total strains)		
	MIC_50_	MIC_90_	Range	MIC_50_	MIC_90_	Range
Amoxicillin	≥32	≥32	16 - ≥ 32	≥32	≥32	≥32
Piperacillin-Tazobactam	≥128	≥128	≥128	≥128	≥128	≥128
Ceftazidime	≥64	≥64	>4 - ≥ 64	≥64	≥64	≥64
Cefotaxime	≥64	≥64	8 - ≥ 64	≥64	≥64	≥64
Cefepime	≥64	≥64	>4 - ≥ 64	≥64	≥64	≥64
Imipenem	>16	>16	>8 - ≥ 16	≥16	≥16	≥16
Meropenem	≥16	≥16	≥16	≥16	≥16	>8 - ≥ 16
Ertapenem	≥8	≥8	4 - ≥ 8	>1	2	>1 - 2
Fosfomycin	64	≥256	≤16 - ≥ 256	≤16	≤16	≤16
Amikacin	32	≥64	≤2 - ≥ 64	≤2	≤2	≤2
Gentamicin	8	≥16	≤1 - ≥ 16	2	2	≤1- 2
Ciprofloxacin	≥4	≥4	>1 - ≥ 4	≥4	≥4	≥4
Trimethoprim-Sulfamethoxazole	≤20	≥320	≤20 - ≥ 320	≥320	≥320	≤20 - ≥ 320
Colistin	≤0,5	≤0,5	≤0,5 - 125	≤0,5	≤0,5	≤0,5

^a^MIC, minimal inhibitory concentration

### Molecular analysis of antimicrobial resistance genes of CRE isolates

PCR and sequence analysis of carbapenemase genes identified *bla*_VIM-1_ in 5 *E.coli* isolates, *bla*_KPC-2_ in 42 *K. pneumoniae* and 1 *R. planticola* isolates*,* and *bla*_KPC-3_ in 9 *K. pneumonia* isolates. No carbapenemase genes were identified in 1 *R. ornithinolytica*, 1 *E. coli*, and 1 *E. aerogenes* isolate (Figs. 1 and 2 and Additional file 1: Table S1). Also, *bla*_CTX-M-1 group_ gene was amplified from all 42 ESBL-producing *K. pneumoniae* isolates; *armA* gene was amplified from all 38 *K. pneumoniae* isolates resistant to aminoglycosides but not from 13 *K. pneumoniae* isolates susceptible to aminoglycosides (Additional file 1: Table S1).

### Molecular epidemiology of CRE isolates in the USL Valle d’Aosta

To investigate whether the isolation of CRE during the study period was caused by the spread of epidemic clones, all 60 CRE isolates were genotyped. Molecular typing by PFGE identified four major PFGE types in 51 *K. pneumoniae* isolates, which we categorized from A to D. Of the 42 *K. pneumoniae* isolates assigned to major PFGE type A, 17 isolates showed an identical macrorestriction pattern (PFGE type A), while 25 strains differed in the migration of one or two DNA fragments and were classified into subtypes A1-A5 (Fig. 1). PFGE types E and F were assigned to *R. ornithinolytica* and *R. planticola* isolates, respectively (data not shown). Also, PFGE analysis identified identical macrorestriction pattern (PFGE type “a”) in 5 *E. coli* isolates and PFGE types “b” and “c”, which differed in the migration of more than 3 bands, in 1 *E. coli* and 1 *E. aerogenes* isolate, respectively (Fig. 2). Genotype analysis using MLST identified ST101 for *K. pneumoniae* isolates assigned to PFGE subtypes A1-A5 and a novel ST1789 for *K. pneumoniae* isolates assigned to PFGE type A. Because ST101 and ST1789 were single locus variants, they were clustered in the same clonal complex (CC) 101 by eBURST analysis. On the contrary, MLST identified ST512 for *K. pneumoniae* isolates assigned to PGFE types B and C and ST405 for *K. pneumoniae* isolates assigned to PGFE type D. ST512 and ST405 differed by at least two loci from all other profiles found in the population under analysis and were regarded as singletons (Fig. 1). Based on all the above data, four distinct epidemic genotypes were identified in *K. pneumoniae* isolates, which we named ST1789/A, ST101/A1-A5, ST512/B-C, and ST405/D, respectively. MLST analysis identified ST131 for all *E. coli* isolates assigned to PFGE type “a” and subtype “a1”, which we named ST131/a epidemic genotype (Additional file 1: Table S1). All *K. pneumoniae* isolates assigned to ST101/A1-A5 and ST1789/A epidemic genotypes were ESBL producers and carried *bla*_CTX-M-1 group_ gene. Also, 23 of 25 *K. pneumoniae* isolates assigned to ST101/A1-A5 epidemic genotype, 8 of 17 *K. pneumoniae* isolates assigned to ST1789/A epidemic genotype, and all *K. pneumoniae* isolates assigned to ST512/B-C epidemic genotype carried *armA* gene and were resistant to aminoglycosides (Tables S1). Four *K. pneumoniae* isolates assigned to ST101/A1-A5 epidemic genotype were resistant to colistin (Additional file 1: Table S1).

To further study the mechanism responsible for carbapenem resistance, we asked whether carbapenem resistance might have been transferred through conjugation. Resistance to imipenem and meropenem (MIC ≥16 mg/l) and ESBL activity along with *bla*_KPC-2_ and *bla*_CTX-M-group 1_ was transferred from *K. pneumoniae* isolates assigned to genotypes ST1789/A and ST101/A1 to *E. coli* J53 strain at frequencies ranging from 8.3 × 10^−4^ to 1.4 × 10^−6^ cfu/recipient cells. Also, resistance to imipenem and meropenem (MIC ≥16 mg/l) along with *bla*_KPC-3_ or *bla*_VIM-1_ was transferred from *K. pneumoniae* isolates assigned to genotypes ST512/B, ST512/C, ST405/D and *E. coli* isolate assigned to genotype ST131/a, respectively, to *E. coli* J53 at frequency ranging from 2.5 × 10^−2^ to 2.5 × 10^−7^ cfu/recipient cells. All transconjugants were resistant to sodium-azide and showed a PFGE profile identical with that of the recipient strain (Table 2). Also, we asked whether conjugative plasmids were responsible for the horizontal transfer of carbapenem resistance. PCR-based replicon typing identified FII replicon and IncFII incompatibility group plasmid/s in ESBL positive and carbapenem-resistant *K. pneumoniae* isolates and transconjugants producing *bla*_KPC-2_ and *bla*_CTX-M-group 1_ genes, while FIIk replicon and IncF incompatibility group plasmid/s in *K. pneumoniae* isolates and transconjugants producing *bla*_KPC-3_ gene. Moreover, the *E. coli* donor strain expressing *bla*_VIM-1_ gene and its transconjugant were shown to carry plasmid/s with N replicon and IncN incompatibility group (Table 2).

**Table 2:** Conjugative transfer of carbapenem resistance in *Enterobacteriacea*

Strain number	Donor Genotype^a^	Transconjugant Genotype^a^	Frequency of transfer^b^	Replicon typing^c^	ESBL^d^	Carbapenemase
5:J53	ST1789/A	ST10/d	8.3×10^−4^	FII	CTX-M-1 group	KPC-2
8:J53	ST101/A1	ST10/d	1.4 × 10^−6^	FII	CTX-M-1 group	KPC-2
2:J53	ST512/B	ST10/d	6.8 × 10^−5^	FIIk	-	KPC-3
12:J53	ST512/C	ST10/d	2.5 × 10^−7^	FIIk	-	KPC-3
24:J53	ST405/D	ST10/d	1.6 × 10^−5^	FIIk	-	KPC-3
6:J53	ST131/a	ST10/d	2.5 × 10^−2^	N	-	VIM-1
J53	ST10/d	ST10/d	-	-	-	-

^a^The genotypes of the strains were identified by MLST sequencing types (STs) and PFGE types. Different MLST schemes were used for K. *pneumoniae and E. coli* as described in the methods section

^b^Mating rate was calculated as frequency of carbapenem resistant transconjugants for recipient cell resistant to sodium-azide in filter mating experiments

^c^Replicon typing was performed as describe in the materials and method section

^d^ESBL (extended-spectrum beta-lactamase)

**Fig. 3:**
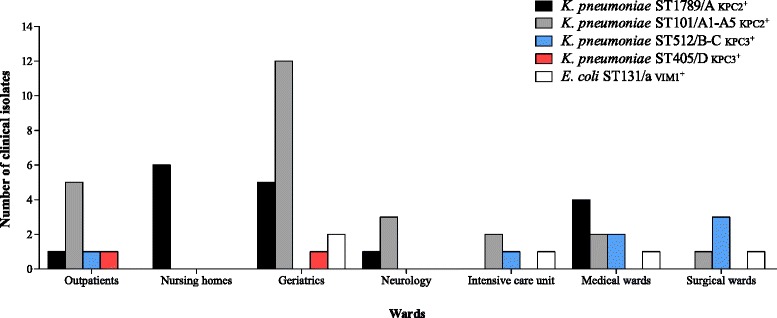
Molecular epidemiology of CRE in the USL Valle d’Aosta. Circulation of CRE epidemic genotypes in the USL Valle d’Aosta, from November 2013 to August 2014. The number of CRE clinical isolates and wards are shown

Molecular epidemiology of CRE isolates showed that the increase of CRE was caused by the spread of KPC-2 producing *K. pneumoniae* ST101/A1-A5 and ST1789/A epidemic genotypes, which belonged to the same CC101. In particular, 17 KPC-2 producing *K. pneumoniae* assigned to ST1789/A were isolated from 17 patients; 25 KPC-2 producing *K. pneumoniae* assigned to ST101/A1-A5 genotype were isolated from 21 patients, 3 patients showing repetitive isolation of *K. pneumoniae* ST101/A1-A3, one of which having isolation of *K. pneumoniae* ST101/A1-A3 in neurology ward and one month later as outpatient. Also, 2 patients showed isolation of ST1789/A and ST101/A2-A3. Seven KPC-3 producing *K. pneumoniae* ST512/B-C, 2 KPC-3 producing *K. pneumoniae* ST405/D, and 5 VIM-1 producing *E. coli* ST131/a were isolated from 7, 2, and 5 patients, respectively. Two different patients showed the isolation of ST512/B1 and ST405/D, and ST101/A1 and ST131/a (Fig. 1). As shown in Fig. 3, KPC-2 producing *K. pneumoniae* ST1789/A were isolated from 6, 5, 4 and 1 patients in nursing homes, geriatrics, medical wards, neurology, respectively, and 1 outpatient; KPC-2 producing *K. pneumoniae* ST101/A1-A5 were isolated from 12, 3, 2, 2, and 1 patients in geriatrics, neurology, intensive care unit, medical and surgical wards, respectively and in 5 outpatients (Fig. 3). KPC-3 producing *K. pneumoniae* ST512/B-C, ST405/D, and VIM-1 producing *E. coli* ST131/a epidemic genotypes were isolated from 2 patients in intensive care unit and 5 and 4 patients in medical and surgical wards, respectively; KPC-3 producing *K. pneumoniae* ST405/D and VIM-1 producing *E. coli* ST131/a genotypes were isolated from 3 patients in geriatrics and 2 outpatients, respectively (Fig. 3).

## Discussion

In the present report, we studied the molecular epidemiology and the genetic basis of carbapenem resistance in CRE isolates in the USL Valle d’Aosta between November 2013 and August 2014. In accordance with previous data [1–7, 9, 10], the spread of CRE in Valle d’Aosta region was caused by the selection of few epidemic distinct genotypes of KPC-2 producing *K. pneumoniae*, KPC-3 producing *K. pneumoniae*, or VIM- producing *E. coli*. The majority of the isolates (42 of 60) were assigned to *K. pneumoniae* ST101/A1-A5 and ST1789/A genotypes, which belonged to the same CC101 and carried *bla*_KPC-2_ into IncFII conjugative plasmid/s. KPC-2 producing *K. pneumoniae* ST101/A1-A5 were isolated from patients in geriatrics, neurology, other medical wards, and from outpatients; KPC-2 producing *K. pneumoniae* ST1789/A were isolated from patients in geriatrics, medical wards, nursing homes, and one outpatient. The isolation of identical genotypes of CRE in long-term care facilities and adjacent hospital wards is in agreement with a previous study showing the isolation of identical carbapenemase among *Enterobacteriaceae* in an Italian tertiary-care hospital and adjacent long-term care facilities [8]. KPC-2 producing *K. pneumoniae* ST101 has already been reported as sporadic isolation in Brazil [38] and sporadic or epidemic isolation in Italy [9, 39–41], while KPC-2 producing *K. pneumoniae* ST1789 and nosocomial outbreak caused by KPC-2 producing *K. pneumoniae* ST101 and ST1789 belonging to CC101 were described for the first time herein. Also, sporadic isolation of KPC-3 producing *K. pneumoniae* ST101 has been reported in Southern Italy [42], thus suggesting that *bla*_KPC-2_ might have been acquired through horizontal gene transfer. In further support of this hypothesis, we demonstrated herein that *K. pneumoniae* ST101 and ST1789 isolates carried *bla*_KPC-2_ into IncFII conjugative plasmid/s. We also observed the spread of KPC-3 producing *K. pneumoniae* ST512/B and ST512/C genotypes. Because *K. pneumoniae* ST512 is a single locus variant of ST258, KPC-3 producing *K. pneumoniae* ST512 isolates can be assigned to the multidrug-resistant high risk CC-258, which was responsible of several epidemics in Europe and Italy [6, 9, 11, 42]. Our data showed also the spread of VIM-1 producing *E. coli* ST131 among CRE isolates. This is consistent with previous studies showing the emergence of hypervirulent *E. coli* ST131 clonal lineage [6] and VIM-like producing CRE in Europe and Northern Italy [4, 6–8, 10].

Several studies demonstrate that epidemic clones of CRE are selected in the community and hospital setting because of their multiple antimicrobial resistances [1–11]. The presence of carbapenemases and the acquisition of carbapenemases through horizontal gene transfer have been frequently reported [1–7]. Accordingly, our data showed that carbapenem resistance, along with *bla*_KPC-2_, *bla*_KPC-3_ and *bla*_VIM-1_ carbapenemase genes carried by conjugative plasmids assigned to IncFII, IncF, and IncN incompatibility groups, respectively, was transferred by filter mating experiments. Interestingly, *bla*_KPC-3_ carried by conjugative plasmid/s assigned to IncF incompatibility group was found in both *K. pneumoniae* ST512 and ST405 epidemic genotypes isolated in the study. Our data are in agreement with previous studies showing *bla*_VIM-1_ carbapenemase into conjugative plasmids assigned to IncN incompatibility group [43], *bla*_KPC-2_ into conjugative plasmids assigned to IncFII incompatibility group [5], *bla*_KPC-3_ into conjugative plasmids assigned to IncF incompatibility group [37]. However, the presence of *bla*_KPC-2_ carbapenemase into conjugative plasmids assigned to IncF incompatibility group has been described also [41]. The above all data suggest that conjugation was responsible for carbapenem resistance acquisition in CRE included in the study. The analysis of antimicrobial resistance profiles of CRE epidemic genotypes showed that they were microbial-drug resistant. In particular, we identified CTX-M1 group ESBL in KPC-2 producing ST101 and ST1789 *K. pneumoniae* isolates and corresponding transconjugants. In agreement with our data, the coexistence of KPC-2 carbapenemase and CTX-M-15 group 1 ESBL has been demonstrated [12–14]. Also, all 7 *K. pneumoniae* isolates assigned to ST512, 23 out of 25 isolates assigned to ST101, and 8 out 17 isolates assigned to ST1789 epidemic genotypes were resistant to aminoglycosides and showed the presence of *armA* gene (Additional file 1: Table S1). This is consistent with previous studies showing the isolation of extensively drug-resistant ArmA-and KPC-2-producing ST101 *K. pneumoniae* isolates in Italy [40, 41]. An interesting finding of our study was the occurrence of colistin resistance in four KPC-2 producing *K. pneumoniae* isolates assigned to ST101/A2-A4 genotypes. Our data are in agreement with recent studies showing the emergence of colistin resistance in KPC-2 producing *K. pneumoniae* isolates from the Netherlands [44] and KPC-2 or KPC-3 producing *K. pneumoniae* isolates from Italy [42, 45, 46]. Because KPC-2 or KPC-3 producing colistin resistant *K. pneumoniae* isolates from Italy and the Netherlands belong to ST258 or ST512 single locus variant [42, 44–46], the data shown herein suggest that colistin resistance may develop in different genetic backgrounds.

## Conclusions

The spread of CRE in Valle d’Aosta region was caused by the selection of KPC-2 producing *K. pneumoniae* ST1789 and ST101 isolates, which were single locus variants and could be assigned to CC101. KPC-2 producing *K. pneumoniae* CC101 were isolated from patients in geriatrics and other hospital medical wards and in outpatients in the USL Valle d’Aosta. KPC-3 producing *K. pneumoniae* ST512 and ST405, and VIM-1 producing *E.coli* ST131 were isolated also. Carbapenem resistance, along with *bla*_KPC-2_, *bla*_KPC-3_ and *bla*_VIM-1_ carbapenemase genes, was transferred by conjugative plasmids assigned to IncFII, IncF, and IncN incompatibility groups, respectively, in filter mating experiments. The emergence of colistin resistance was observed in KPC-2 producing *K. pneumoniae* ST101 isolates.

## Additional file

Additional file 1: Table S1. Phenotypic and genotypic features of *Enterobacteriaceae* included in the study. (XLSX 19 kb)

## Abbreviations

BA: Bronchial aspirate
CC: Clonal complex
CRE: Carbapenem resistant *Enterobacteriaceae*
DL: Drainage liquid
EMO: Blood culture
ESBL: Extended-spectrum beta-lactamase
MIC: Minimal inhibitory concentration
MLST: Multi-locus sequence typing
PF: Peritoneal fluid
PFGE: Pulsed-field gel electrophoresis
RS: Rectal swab
ST: Sequencing type
UC: Urine culture
WS: Wound swab
